# Health literacy and mental health: a national cross-sectional inquiry

**DOI:** 10.1038/s41598-024-64656-7

**Published:** 2024-06-13

**Authors:** Aliasghar Haeri-Mehrizi, Samira Mohammadi, Shahram Rafifar, Jila Sadighi, Ramin Mozaffari Kermani, Rahele Rostami, Akram Hashemi, Mahmoud Tavousi, Ali Montazeri

**Affiliations:** 1 Health Metrics Research Center, Iranian Institute for Health Sciences Research, ACECR, Tehran, Iran; 2 Department of Medical Laboratory Science, College of Science, Knowledge University, Erbil, 44001 Iraq; 3 Health Education and Promotion Office, Deputy of Health, Ministry of Health and Medical Education, Tehran, Iran

**Keywords:** Psychology, Public health

## Abstract

Health literacy and mental health are among the most important issues in the modern health and era of public health. This study aimed to investigate the association between health literacy and mental health status. This was a national cross-sectional study that was conducted in Iran. A sample of literate individuals aged 18–65 was entered into the study using multistage sampling. The data were collected by the Health Literacy Instrument for Adults (HELIA) and the 12-item General Health Questionnaire (GHQ-12). Logistic regression and path analysis were used for data analysis. A total of 20,571 individuals completed the questionnaires. The mean(± SD) age of participants was 34.9(± 11.8) years old, 51% were female, and 38.1% had higher education. The mean(± SD) health literacy score was 68.3(± 15.2), and 29.8% of the respondents reported some mental health problems. Logistic regression analysis showed that limited health literacy was associated with poor mental health status (OR 2.560, 95% CI 2.396–2.735, P = 0.001). The path analysis showed that an increase in health literacy could reduce psychological and social dysfunction (the effect of health literacy on reducing psychological distress is more profound). It is recommended to carry out interventions that strengthen adult’s cognitive and communication skills to improve their ability to access and use health information to make healthy choices.

## Introduction

Health literacy is an important concept in public health and is described as individuals’ capacity to use health information and services to maintain and improve health^1^. Poor health literacy was also related to many adverse outcomes such as less use of preventive services^2^, lower adherence to medical prescription^3^, increased risk of hospitalization^4,5^, causing higher healthcare costs^6^, and increased risk of many other ill health conditions, including mental health disorders^7^.

The World Health Organization (WHO) defines mental health as ‘a state of mental well-being that enables people to cope with the stresses of life, realize their abilities, learn well and work well, and contribute to their community’^8^. According to WHO's estimation, in 2019, one in every eight people, or 970 million people worldwide, were suffering from a mental disorder^9^. Of these 80% of those who suffer, reside in low- and middle-income countries^10^. The disability-adjusted life-years (DALY) of mental disorders in the Middle East and North African countries are estimated to be 1990.5 per 100,000 people in 2019^11^. In Asia, the trend of mental disorders increased from 377.9 million cases in 1990 to 555.4 million cases in 2019^12^. In 2020, the prevalence of mental disorders among the general population in Iran was 29.7%^13^.

Studies suggest that understanding various mental health treatments and patient education materials often requires a high level of health literacy in general and mental health literacy in particular^14–16^. Low literacy is a global issue^17^. Comparing health literacy levels across populations is challenging, but national studies can provide useful insight into the topic. The European Health Literacy Survey (HLS-EU) found that 47.6% of respondents across eight countries had inadequate or problematic health literacy. In the US it was reported that 36% of adults have limited health literacy. In Japan, 49.9% of the population was found to have inadequate health literacy^18^. A study in Iran showed that 44% of adults had limited health literacy^19^.

**Figure 1:**
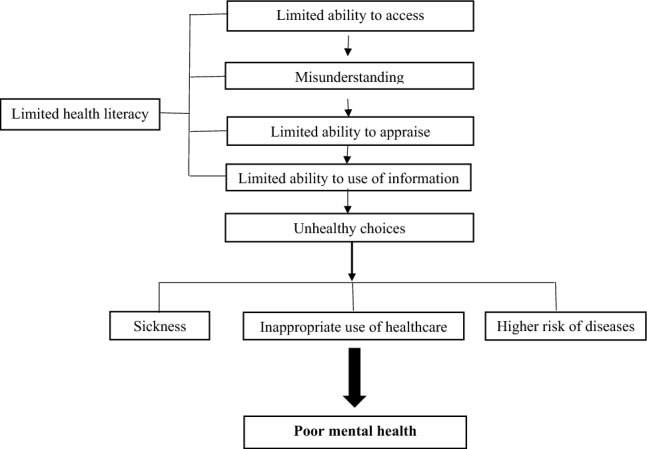
A schematic view of a relationship between limited health literacy and poor mental health.

A limited level of health literacy restricts the ability to access, understand, appraise, and utilize health-related information for decision-making^20^. It thus leads to less ability and skills in healthy choices that in turn might contribute to a poor level of mental health^21^. Unhealthy choices can put people at risk of disease, make them sick, or affect their use of healthcare. The previously established relationship between limited health literacy and poor mental health, anxiety^22^, and depressive symptoms^23–26^. A schematic view of the mechanism of a relationship between limited health literacy and poor mental health is provided in Fig. 1.

However, recently numerous studies investigated the relationship between mental health literacy and mental health^27–29^, but only a few studies have explored the association between functional health literacy and mental health. Even these studies had limited objectives or focused on specific populations^7,23–26,30,31^. As such, it seems that evidence on the relationship between health literacy and mental health remains insufficient. Thus, this study aimed to investigate the association between health literacy and mental health among Iranian adults.

## Methods

### Study design and participants

A national cross-sectional study was conducted on a random sample of Iranian adults from all provinces of Iran. As such the capital of each province and a city (drawing from the list of cities in each province) were selected. The inclusion criteria were 18 to 65 years old, Iranian nationality, residing in urban areas, and able to read and write. The exclusion criteria included individuals who were outside the age range, and people with mental disabilities. No other restrictions were implemented. The study was conducted following the Helsinki Declaration.

### Instruments

The data were collected by using two questionnaires:

1. The Health Literacy Instrument for Adults (HELIA): The HELIA is a validated 33-item questionnaire tapping into five dimensions: access to health information, reading, understanding, appraisal, and behavioral intention. Each item is rated on a 5-point Likert scale giving a total score ranging from zero (the worst) to 100 (the best). The scores could be categorized as follows: ‘inadequate: 0–50’, ‘problematic’: 50.1–66 (which together also can indicate ‘limited’ health literacy: 0–66), ‘sufficient’:66.1–84 and ‘excellent: 84.1–100 (which together also can indicate ‘desirable’ health literacy: 66.1–100). The original version indicated satisfactory reliability and validity, as evidenced by Cronbach’s alpha coefficients ranging from 0.72 to 0.89^32,33^. However, for the current study, Cronbach alpha coefficient was 0.93.

2. The 12-item General Health Questionnaire (GHQ-12): The General Health Questionnaire (GHQ) is a measure of current mental health status^34^ that has been extensively used in different settings and different cultures^35–37^. The questionnaire asks whether the respondent has recently experienced a particular symptom or behavior. Each item is rated on a four-point scale (less than usual, no more than usual, rather more than usual, or much more than usual); for example, when using the GHQ-12, it gives a total score of 36 or 12 based on the selected scoring methods. One common scoring method is the bi-model (0-0-1-1). Since the GHQ-12 is brief, simple, and easy to complete, its application in research settings as a screening scale is well documented, and this version of the questionnaire was used. The Persian version of GHQ-12 was validated with 12 items and two dimensions including 'psychological distress' and 'social dysfunction'. The reliability analysis was reported to be satisfactory (Cronbach's alpha coefficient = 0.87). Convergent validity indicated a significant negative correlation between the GHQ-12 and global quality of life scores as expected (r = −0.56, P < 0.0001)^38^. The Cronbach's alpha coefficient for the GHQ-12 in this study was 0.81. According to the previous study, respondents who scored greater than four were considered to ‘have mental health problems', and those who scored equal or less than four were considered to ‘have no mental health problems’^39^.

### Procedure

At the beginning of the data collection, people were asked for consent. After they accepted to take part in the survey, the interviewers asked the questions one by one and filled in the demographic details and the health literacy and mental health questionnaire. All interviewers underwent specific training tailored to this study, ensuring adherence to ethical guidelines and consistency in data collection.

### Sample size and sampling

The sample size was calculated using the following formula:

$$n = \frac{{z^{2} a/2 p \cdot q}}{{\left( {rp} \right)^{2} }}$$

Regarding previous national studies on the prevalence of mental disorders in Iran, 21% in 1999 and 20.9% in 2004, and considering the proportion (p) as 0.2, a relative error (r) as 0.03 and α = 0.05 and applying design effect of 1.33, a sample size of 19,900 was estimated. However, in practice, 20,571 Iranian adults were entered into the study. Each household was considered as the sampling unit. Within each household, an adult was included in the study.

### Statistical analysis

Data were explored using descriptive statistics, including frequency, percentage, mean and standard deviation. Logistic regression analyses were performed to assess the relationship between mental health and independent variables. As such, mental health was considered as outcome variables and participants’ health literacy, age, gender, educational level and employment treated as independent variables. The results were expressed as odds ratio and 95% confidence intervals (CI). A significant level was set at P < 0.05. The path analysis was conducted to investigate the contribution of health literacy to mental health. All statistical analyses were carried out using SPSS software version 23 as well as AMOS 23 software for path analysis.

### Ethics declarations

The Iranian Academic Center for Education, Culture, and Research (ACECR) approved the study (IR.ACECR.IBCRC.REC.1394.44).

### Consent to participate

Due to the study design, all participants gave their verbal informed consent.

## Results

### Participant

A total of 20,571 individuals participated in the study. The mean age of the participants was 34.9 ± 11.8(± SD) years, 51% were female and 38.1% had higher education. The socio-demographic characteristics of the participants are shown in Table 1.

**Table 1:** Demographic characteristics of participants (n = 20,571).

	Total (n = 20,571)	Male (n = 10,135)	Female (n = 10,436)	P-value
	No. (%)	No. (%)	No. (%)	
Age (years)				P < 0.001
18–24	4597 (22.4)	2321 (22.9)	2263 (21.7)	
25–34	6977 (33.9)	3315 (32.7)	3662 (35.1)	
35–44	4353 (21.2)	2110 (20.8)	2243 (21.5)	
45–54	3043 (14.7)	1509 (14.9)	1534 (14.7)	
55–68	1601 (7.8)	871 (8.6)	730 (7.0)	
Education level (years)				P < 0.001
Up to 5	2119 (10.3)	749 (7.4)	1369 (13.1)	
6–9	3024 (14.7)	1469 (14.5)	1545 (14.8)	
10–12	7591 (36.9)	3638 (35.9)	3955 (37.9)	
Higher than 12	7837 (38.1)	4279 (42.2)	3567 (34.2)	
Employment				P < 0.001
Employed	8036 (39.1)	6402 (63.2)	1634 (15.7)	
Housewife	6574 (32.0)	0 (0.0)	6574 (63)	
Retired	1229 (6.0)	993 (9.8)	236 (2.3)	
Student	3048 (14.8)	1653 (16.3)	1395 (13.4)	
Unemployed	1684 (8.2)	1087 (10.7)	597 (5.7)	

### Health literacy and mental health

The mean health literacy score was 68.3 ± 15.2 (± SD). In addition, the results showed that 29.8% of the respondents reported poor mental health (according to the GHQ-12 cut of point 4 ≥). The detailed results are presented in Table 2.

**Table 2:** Frequency distributions of health literacy and mental health (n = 20,571).

	Male (n = 10,135)	Female (n = 10,436)	Total (n = 20,571)
	No. (%)	No. (%)	No. (%)
*Health literacy*			
Access to health information			
Adequate	5227 (51.4)	5438 (52.1)	10,665 (51.8)
Limited	4908 (48.6)	4998 (47.9)	9906 (48.2)
Reading			
Adequate	5424 (53.5)	5976 (57.3)	11,400 (55.4)
Limited	4711 (46.5)	4460 (42.7)	9171 (44.6)
Understanding			
Adequate	6795 (67.0)	7493 (71.8)	14,288 (69.5)
Limited	3340 (33.0)	2943 (28.2)	6283 (30.5)
Appraisal			
Adequate	4897 (48.3)	5144 (49.3)	10,041 (48.8)
Limited	5238 (51.7)	5292 (50.7)	10,530 (51.2)
Behavioral intention			
Adequate	5759 (56.8)	6385 (61.2)	12,144 (59.0)
Limited	4376 (43.2)	4051 (38.8)	8427 (41.0)
Total health literacy			
Adequate	5610 (55.4)	6207 (59.5)	11,817 (57.4)
Limited	4525 (44.6)	4229 (40.5)	8754 (42.6)
Mental health			
Good	7277 (71.8)	7170 (68.7)	14,447 (70.2)
Poor	2858 (28.2)	3266 (31.3)	6124 (29.8)

### The relationship between mental health and independent variables

The results obtained from logistic regression analysis showed that poor mental health was associated with limited health literacy (OR 2.560, 95% CI 2.396–2.735, p = 0.001), gender (OR for female: 1.172, 95% CI 1.067–1.287, P = 0.001) and employment (OR for unemployed: 1.967, 95% CI 1.746–2.215, P = 0.001). However, better mental health was associated with higher age (OR 0.994, 95% CI 0.991–0.998, P = 0.002), and higher education (OR 0.961, 95% CI 0.951–0.970, P = 0.001). The results are shown in Table 3.

**Table 3:** The association between health literacy and poor mental health obtained from logistic regression analysis (n = 19,903).

	Univariate		Multivariate*	
	OR (95% CI)	P value	OR (95% CI)	P value
Age(years)	0.997 (0.994–0.999)	0.016	0.994 (0.991–0.998)	0.002
Gender				
Male	1.0 (ref)		1.0 (ref)	
Female	1.160 (1.091–1.234)	0.001	1.172 (1.067–1.287)	0.001
Education (years)	0.941 (0.933–0.948)	0.001	0.961 (0.951–0.970)	0.001
Employment				
Employed	1.0 (ref)		1.0 (ref)	
Housewife	1.374 (1.274–1.481)	0.001	1.076 (0.961–1.204)	0.205
Retired	0.821 (0.707–0.953)	0.001	0.892 (0.754–1.055)	0.180
Student	1.071 (0.927–1.181)	0.164	0.998 (0.891–1.117)	0.971
Unemployed	2.201 (1.968–2.462)	0.001	1.967 (1.746–2.215)	0.001
Health literacy				
Adequate	1.0 (ref)		1.0 (ref)	
Limited	2.721 (2.555–2.899)	0.001	2.560 (2.396–2.735)	0.001

*Adjusted for age, gender, education and employment.

### A path analysis of the relationship between health literacy and mental health

**Figure 2:**
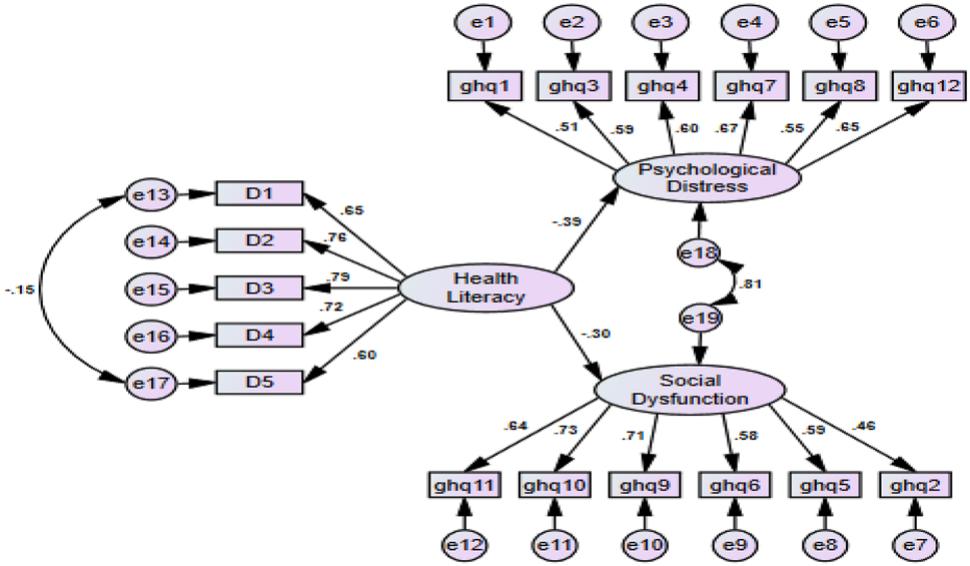
A path analysis of the relationship between health literacy and mental health.

The results obtained from path analysis showed that a higher level of health literacy could reduce psychological distress and social dysfunction. The details are shown in Fig. 2. Similarly, the findings showed satisfactory fit indexes for the model as expected. The model fit indexes are shown in Table 4.

**Table 4:** The goodness of fit indexes results from path analysis.

	Obtained indexes	Cut of values
GFI	.945	> 0.9
AGFI	.926	> 0.9
NFI	.918	> 0.9
RFI	.903	≥ 0.90
IFI	.919	≥ 0.90
TLI	.904	≥ 0.95
CFI	.919	≥ 0.95
RMSEA	.044	≤ 0.08

*AGFI* adjusted GFI, *GFI* Goodness-of-Fit Index, *NFI* Normed Fit Index, *RFI* Relative Fit Index, *IFI* Incremental Fit Index, *TLI* Tucker–Lewis Index, *CFI* Comparative Fit Index, *PNFI* Parsimonious Normed Fit Index, *RMSEA* root mean square error of approximation.

## Discussion

This national study examined the association between health literacy and mental health in 20,571 adults aged 18 to 65 in Iran. The findings showed that limited health literacy was associated with poor mental health even after adjustment for demographic and socioeconomic factors, including age, gender, education, and employment status.

Many studies showed that lower health literacy was related to poorer health outcomes^4–6,40^. However, concerning the relationship between health literacy and mental health, most studies are focused on mental health and mental health literacy^27–29^. As such a few existing studies have confirmed that there is a relationship between health literacy and mental disorders, showing a clear link between low health literacy and mental health problems^23,25,26,30,31^. To the best of our knowledge, these studies are specific in their scope, and we have not been able to find a national study that examined this issue. A study in the US suggested that individuals with certain mental illnesses and lower functioning may have more difficulty understanding health information and have limited numerical literacy^41^. A study showed that adults with higher functional and communicative health literacy had lower mental health stigma and aversion to mental health help-seeking^42^. One might argue that adequate health literacy could lead to healthy choices and better achievements in life, thus improving mental health. Longitudinal studies would be essential for establishing the causal relationship between these variables.

The findings from the current study indicated that female gender, younger age, lower educational level, and unemployment status independently were associated with poor mental health It is possible to argue that was not health literacy that caused poor mental health; rather, social determinants are responsible for such observation. In other words, lower socioeconomic status leads to lower health literacy, and lower health literacy leads to poorer mental health. In this respect, it is possible to say that the relationship between health literacy and mental health is not direct and casual. Instead, it emerges due to poor socioeconomic status or lower social class. This argument is most important in low- and middle-income countries because people in these regions face specific challenges such as financial challenges, poverty, and inadequate services^10^. The findings of the integrative review confirmed that health literacy is one of the fundamental potential mediating factors that link socioeconomic status and health. Enhancing the level of health literacy in the population or making health services more accessible to people with low health literacy may be a means to reach a greater equity in health^43,44^.

### Strengths and limitations

The relatively large sample size and people with different socio-economic backgrounds were the study strengths. In addition, data collection was conducted by trained interviews. However, this study was cross-sectional; thus, the findings do not convey the casual association. Longitudinal studies are needed to explore the causal relationships. Although we carried out a logistic regression analysis, the exact mechanism for the relationship between health literacy and mental health remains to be examined further. Finally, one should note that the ethics approval for this study was obtained approximately 8 years ago, but due to some logistic problems, the study commenced with a delay. Perhaps the study needs to be replicated in due course to provide more updated information on the topic.

## Conclusion

The study findings suggest that cognitive and social skills in processing health information could positively affect mental health. It is recommended to carry out interventions that strengthen adult’s cognitive and communication skills to improve their ability to access and use health information to make healthy choices.

## Data Availability

The datasets used and analyzed during the current study are included in this article.
